# Analysis of inner and outer retinal layers using spectral domain optical coherence tomography automated segmentation software in ocular hypertensive and glaucoma patients

**DOI:** 10.1371/journal.pone.0196112

**Published:** 2018-04-19

**Authors:** Pilar Cifuentes-Canorea, Jorge Ruiz-Medrano, Rosa Gutierrez-Bonet, Pablo Peña-Garcia, Federico Saenz-Frances, Julian Garcia-Feijoo, Jose Maria Martinez-de-la-Casa

**Affiliations:** 1 Servicio de Oftalmología, Hospital Clínico San Carlos, Departamento de Oftalmología, Facultad de Medicina, Universidad Complutense de Madrid and Instituto de Investigación Sanitaria del Hospital Clínico San Carlos, Madrid, Spain; 2 Hospital Puerta de Hierro, Madrid, Spain; 3 Division of Ophtalmology, Miguel Hernandez University, Alicante, Spain; Universidade Federal do Rio de Janeiro, BRAZIL

## Abstract

**Objective:**

To analyse the morphological features and diagnostic ability of eight macular retinal layers using a new segmentation software Heidelberg's Spectralis Optical Coherence Tomography (SD-OCT) in healthy, ocular hypertensive and primary open angle glaucoma patients.

**Methods:**

Single-center, cross-sectional, non-interventional study. 193 eyes from 193 consecutive patients (56 controls, 63 ocular hypertensives, 32 early primary open glaucoma patients and 42 moderate-advanced primary open glaucoma patients). Those patients presenting any retinal disease were excluded. Macular segmentation of the retinal layers was automatically performed using the new segmentation Heidelberg's Spectralis OCT software providing measurements for eight retinal layers. The software provides thickness maps divided into nine subfields.

**Results:**

Statistically significant differences in inner layers’ thickness was found between all 4 four groups. Superior and inferior sectors of macular retinal nerve fiber layer; nasal, temporal, superior and inferior sectors of ganglion cell layer and inner plexiform layer were significantly different when comparing ocular hypertensive patients and early glaucoma patients. Areas under the ROC curves for early glaucoma diagnosis were 0.781±0.052 for macular retinal nerve fiber layer outer inferior sector, 0.760±0.050 for ganglion cell layer outer temporal sector, 0.767±0.049 for the inner plexiform layer outer temporal sector and 0.807±0.048 for the combination of all three. No differences were found between groups when considering outer retinal layers.

**Conclusions:**

The automated segmentation software from Heidelberg's Spectralis OCT provides a new diagnostic tool for the diagnosis of ocular hypertensive and glaucoma patients.

## Introduction

Glaucoma is still the second leading cause of blindness worldwide [1]. Its multifactorial etiology leads to a progressive loss of retinal ganglion cells (RCG) [2,3] and a reduction in the patients’ visual field (VF) [4,5].

Diagnostic tools for glaucoma have evolved during the years seeking to learn more about this disease and to improve methods to analyse its progression. Tests performed for the diagnosis of glaucoma include intraocular pressure (IOP) measurement, VF tests [6], stereo and red-free photographs [7] and more recently, structural analysis tests like optical coherence tomography (OCT) [8], which grants a more precise and reliable analysis of the optic nerve and peripapillary retinal nerve fiber layer (pRNFL) [9,10].

The study of macular RNFL (mRNFL) has recently been updated as a result of previous papers by Zeimer et al [11], and has brought forward recent studies that are analysing the macula in search of glaucomatous tissue damage due to the great concentration of RCG at this level [3,12–17]. Recent studies have shown that macular measurements with Spectral Domain (SD) OCT were as good as the pRNFL measurements in detecting glaucoma [18–23].

The introduction of new software tools for macular segmentation for SD-OCT devices such as Heidelberg’s Spectralis (Heidelberg Engineering, Inc., Heidelberg, Germany) enable the identification and measurement of each one of the macular retinal layers [17].

There are many reports that demonstrate the alteration of the inner retinal layer in glaucoma patients. Nevertheless, there is controversy about the damage affecting outer retinal layers [24–30]. The aim of this study is to evaluate the thickness of eight macular layers in normal, ocular hypertensive (OHT) and glaucomatous eyes using the latest version of automated retinal segmentation software from Heidelberg's Spectralis OCT, analysing the variations between groups and evaluating its diagnostic capability.

## Material and methods

This is a cross-sectional observational study performed at the Glaucoma Department of Clinico San Carlos University Hospital, Informed consent was obtained from all the study’s participants and all methods were approved by the Ethics Committee of the Hospital Clinico San Carlos. The study protocol adhered to the Declaration of Helsinki for research involving human subjects. The subjects provided informed consent to participate in the research to study the thickness and morphology of macular retinal layers in normal, OHT and glaucoma subjects. Participation was offered to patients attending our Glaucoma department who met the inclusion criteria and voluntarily agreed to participate. For each patient, one eye was randomly selected for the final analysis.

Inclusion criteria were best-corrected visual acuity of 20/40 or better, refractive error of less than 5 spherical diopters and 2 diopters of cylinder, transparent ocular media (<1 according to the Lens Opacities Classification System III system) [31], open anterior chamber angle, presence of OHT (group 2) or primary open glaucoma (POAG) for groups 3 and 4.

Those patients presenting any retinal disease, surgeries performed during the 3 previous months to the inclusion, ophthalmological or neurological disease history, diabetes or use of medication that could alter VF sensitivity, were excluded.

All subjects underwent a complete review of medical history and ophthalmologic examination, including visual acuity, slit lamp examination, IOP measurement using Goldmann applanation tonometry, central corneal thickness measurement, dilated fundus examination, VF and macular OCT. Patients were classified into 4 groups according to the results of the tests in healthy, OHT, early POAG, and moderate-advanced POAG.

Patients were classified as healthy (Group 1) when they presented IOP lower than 21 mmHg, normal appearance of the optic nerve head (ONH) and normal standard automatic perimetry (SAP) results. They were classified as OHT (Group 2) when they showed a normal appearance of the ONH, an elevated IOP (>21 mmHg) and normal SAP. Patients presenting ONH cupping or damage alongside, alterations in SAP results were classified as glaucomatous and then divided in 2 groups using the scheme proposed by Hodapp et al [32] as early (mean deviation (MD) ≤ -6.00 dB)(Group 3), moderate-advanced glaucoma (MD ≥ 6 dB)(Group 4).

### Visual field

Humphrey VF Analyser implementing a Swedish Interactive Threshold Algorithm Standard strategy (Carl Zeiss Meditec, Dublin, CA, USA) was used. The test was repeated in those cases where fixation losses surpassed 20% or false-positive or false-negative rates surpassed 15%. A normal SAP result was defined as visual field indexes (MD and pattern standard deviation) within 95% confidence limits, with fewer than three non-edge contiguous points within the same hemifield identified as significant (P < 0.05) in the pattern deviation plot, and glaucomatous hemifield test results within normal limits.

### Optical coherence tomography

A ‘‘macular cube” protocol was performed on all subjects. Volumetric retinal scans comprising 25 single horizontal axial scans (scanning area: 666 mm2 centered at the fovea) were obtained using image alignment eye-tracking software (Tru-Track; Heidelberg Engineering. Heidelberg, Germany). The same experienced operator performed all scans. No manual correction was applied to the OCT output. The quality of the scans ranging from 0 (poor) to 40 (high) was assessed before analysis and scans scoring lower than 25 were rejected.

**Fig 1:**
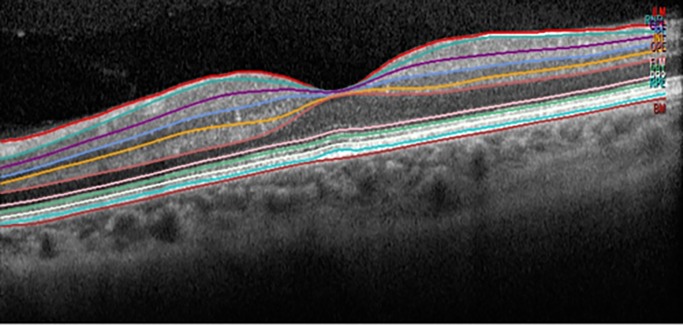
Segmentation of the retinal layers. Single horizontal foveal scans were automatically performed using new prototype software for the Heidelberg's Spectralis OCT Spectralis. Inner limiting membrane (ILM), retinal nerve fiber layer (RNFL), ganglion cell layer (GCL), inner plexiform layer (IPL), inner nuclear layer (INL), outer plexiform layer (OPL), outer nuclear layer (ONL), external limiting membrane (ELM), photoreceptor layer (PRC) and retinal pigment epithelium (RPE), Bruch’s membrane (BM).

Segmentation of the retinal layers in single horizontal foveal scans was automatically performed using the last software version for the Heidelberg's Spectralis OCT, version 5.4b, providing eight measures (Fig 1): mRNFL, ganglion cell layer (GCL), inner plexiform layer (IPL), inner nuclear layer (INL), outer plexiform layer (OPL), outer nuclear layer (ONL), photoreceptors layer (PRC) and retinal pigment epithelium (RPE).

The new software from Spectralis automatically provides thickness maps divided into nine subfields as defined by the Early Treatment Diabetic Retinopathy Study (ETDRS). Inner, intermediate, and outer rings with diameters of 1, 3, and 6 mm respectively, were considered for the analysis. The average of all points within the inner 1-mm radius circle was defined as central foveal thickness (C0). The intermediate ring was divided into four sectors designated as inner superior (S1), inner nasal (N1), inner inferior (I1), and inner temporal (T1); and so was the outer ring, with four sectors designated as outer superior (S2), outer nasal (N2), outer inferior (I2), and outer temporal (T2). The numerical values recorded for each of the nine zones for every layer were used in the analysis.

### Statistical analysis

The program used for the statistical treatment of the data was version 17.0 of SPSS for Windows (SPSS, Chicago, IL). In order to check normality, Kolmogorov-Smirnov test was applied to all data. Comparison between groups was performed using the Student t test when samples were normally distributed, or the Mann-Whitney test whenever parametric statistics were not possible. The level of significance used was p<0.05. We corrected for the effect of multiple comparisons by conducting an a posteriori Bonferroni test by the Bonferroni factor. For the comparison of several independent samples, the Analysis of Variance (ANOVA) or Kruskal-Wallis test were used depending on whether normality could be assumed.

The diagnostic capacity of each variable to differentiate between normal and glaucoma suspect eyes was determined by calculating the area under the receiver operating characteristics (ROC) curve (AUC). The ROC curve shows the trade-off between sensitivity and 1_specificity (false-positive rate). An area under the ROC curve (AUC) of 1.0 represents perfect discrimination, whereas an AUC of 0.5 represents chance discrimination. Differences between the ROC curves were tested to compare AUCs using the Hanley–McNeil method [33].

## Results

The macular area of 193 eyes from 193 patients, 56% males and 44% women, 50.2% left eyes and 49.8% right eyes were analysed using the procedure described before (n1 = 56 healthy, n2 = 63 OHT, n3 = 32 early POAG, n4 = 42 moderate-advanced POAG).

Mean VF MD was 0.51±1.15 for group 2, -4.00±1.00 for group 3 and -9,35±2.88 for group 4. Mean age was 71.85 years old for group 1, 69.46 for group 2, 74.03 for group 3 and 76.46 for group 4. There was no statistical difference regarding age between the first three groups (p = 0.059, Kruskal-Wallis test). However, group 4 was statistically older.

Thickness values for all layers in each of the 9 ETDRS sectors were obtained and represented in S1 File. Values for INL, OPL, ONL, PRC and RPE for each of the four groups are overlapped in every sector, showing no statistically significant differences. On the other hand, mRNFL, GCL and IPL show clear differences between four groups, so their potential discrimination ability was studied in depth.

Differences between these 3 layers were found amongst the groups studied in all sectors with the exception of T1 sector for mRNFL and C0 sector for IPL (Tables 1–3).

**Table 1:** Macular retinal nerve fiber layer analysis.

mRNFL Mean±SD [95% CI]	Healthy controls (group 1)	Ocular hypertensive (group 2)	Early glaucoma (group 3)	Moderate-advanced glaucoma (group 4)	P value for all four groups comparison (Kruskal-Wallis test)	P value for groups 2 and 3 comparison (Mann-Whitney test)
C0	13.35±4.55 [12.75, 15.18]	13.69±4.13 [13.00, 15.13]	12.60±3.78 [1.76, 14.49]	11.89±3.43 [10.93, 13.18]	0.005	0.53
N1	22.53±3.14 [21.89, 23.57]	22.00±4.76 [21.19, 23.65]	21.49±4.39 [20.23, 23.40]	19.57±3.14 [18.78, 20.85]	<0.001	0.231
N2	47.01±7.11 [45.29, 49.10]	44.69±6.16 [42.88, 46.06]	40.75±8.75 [38.03, 44.34]	33.25±6.97 [30.68, 35.26]	<0.001	0.065
S1	27.82±4.80 [26.84, 29.41]	27.04±4.57 [25.97, 28.33]	24.97±4.40 [23.57, 26.74]	22.16±4.16 [21.11, 23.84]	<0.001	0.008
S2	41.72±5.95 [40.18, 43.36]	39.99±6.06 [38.62, 41.67]	35.74±6.74 [33.45, 38.30]	27.18±6.44 [25.22, 29.46]	<0.001	0.002
T1	18.63±2.51 [18.09, 19.44]	17.98±2.23 [17.65, 18.77]	18.22±1.96 [17.58, 18.99]	17.83±2.59 [17.20, 18.90]	<0.355	0.489
T2	21.07±2.66 [20.48, 21.91]	20.64±3.04 [20.17, 21.70]	19.85±2.59 [19.19, 21.06]	18.82±2.34 [18.02, 19.56]	<0.001	0.144
I1	26.82±4.09 [25.90, 28.10]	24.48±3.49 [23.60, 25.36]	22.74±4.09 [21.52, 24.48]	20.20±3.57 [18.96, 21.30]	<0.001	0.040
I2	42.69±6.98 [40.99, 44.73]	39.39±5.19 [37.93, 40.55]	33.07±7.59 [31.05, 36.52]	25.13±6.57 [23.16, 27.48]	<0.001	8·10^−6^

Measurements in μm

SD: Standard Deviation; CI: Confidence Interval; mRNFL: Macular retinal ner

**Table 2:** Ganglion cell layer analysis.

GCL Mean±SD [95% CI]	Healthy controls (Group 1)	Ocular hypertensive (Group 2)	Early glaucoma (Group 3)	Moderate-advanced glaucoma (Group 4)	P value for all four groups comparison (Kruskal-Wallis test)	P value for groups 2 and 3 comparison (Mann-Whitney test)
C0	15.23±5.57 [13.73, 16.74]	16.82±5.69 [15.68, 18.62]	16.19±10.65 [14.04, 21.71]	13.77±5.83 [12.38, 16.20]	0.010	0.346
N1	52.58±7.61 [50.31, 54.42]	51.70±8.19 [49.43, 53.67]	47.10±7.95 [43.82, 49.56]	38.46±8.68 [35.28, 40.99]	<0.001	0.008
N2	40.20±5.28 [38.72, 41.57]	39.93±5.37 [38.58, 41.35]	36.71±5.28 [34.88, 38.69]	31.40±4.99 [29.57, 32.85]	<0.001	0.008
S1	52.50±6.03 [50.70, 53.96]	51.50±7.75 [48.73, 52.74]	46.43±6.66 [43.91, 48.72]	36.22±7.88 [33.28, 38.46]	<0.001	4·10^−4^
S2	32.27±3.73 [31.25, 33.26]	31.02±4.14 [29.95, 32.09]	28.79±3.61 [27.39, 29.99]	23.78±4.08 [22.26, 24.95]	<0.001	0.013
T1	48.44±7.06 [46.38, 50.20]	46.52±7.51 [44.48, 48.36]	40.50±6.65 [38.29, 43.08]	29.66±7.81 [26.70, 31.83]	<0.001	6·10^−5^
T2	38.42±4.29 [37.17, 39.49]	36.37±5.63 [35.01, 37.92]	31.42±4.35 [29.81, 32.94]	24.46±5.36 [22.45, 25.97]	<0.001	1·10^−5^*
I1	51.57±6.63 [49.55, 53.14]	50.11±9.39 [47.02, 51.88]	43.83±8.50 [40.47, 46.60]	34.25±10.15 [30.61, 37.28]	<0.001	2·10^−4^
I2	32.58±4.17 [31.47, 33.73]	32.56±4.49 [31.27, 33.59]	28.87±5.08 [27.20, 30.86]	25.50±3.99 [23.50, 26.13]	<0.001	4·10^−4^

Measurements in μm

SD: Standard Deviation; CI: Confidence Interval; GCL: Ganglion cell layer

**Table 3:** Inner plexiform layer analysis.

IPL Mean±SD [95% CI]	Healthy controls (Group 1)	Ocular hypertensive (Group 2)	Early glaucoma (Group 3)	Moderate-advanced glaucoma (Group 4)	P value for all four groups comparison (Kruskal-Wallis test)	P value for groups 2 and 3 comparison (Mann-Whitney test)
C0	21.87±6.01 [20.64, 23.86]	22.44±4.29 [21.51, 23.72]	22.44±5.80 [21.04, 25.22]	20.72±3.99 [19.59, 22.21]	0.140	0.822
N1	41.64±13.41 [39.53, 46.72]	40.33±4.46 [38.90, 41.20]	38.06±5.23 [36.02, 39.79]	32.74±5.55 [31.05, 34.69]	<0.001	0.051
N2	32.48±7.40 [31.22, 35.18]	32.13±4.13 [31.15, 33.28]	28.72±4.03 [27.24, 30.14]	25.22±3.75 [24.18, 26.66]	<0.001	2·10^−4^
S1	40.66±6.14 [39.41, 42.70]	40.30±5.18 [38.65, 41.32]	36.90±4.30 [35.17, 38.27]	30.98±5.41 [29.30, 32.86]	<0.001	3·10^−4^
S2	27.25±4.16 [26.30, 28.53]	26.48±4.40 [25.51, 27.79]	23.45±2.67 [22.44, 24.37]	20.41±2.85 [19.43, 21.31]	<0.001	4·10^−4^
T1	40.73±5.54 [39.05, 42.02]	38.94±5.09 [37.52, 40.14]	36.40±3.51 [35.11, 37.64]	31.44±4.97 [29.71, 32.98]	<0.001	0.002
T2	34.51±3.29 [33.64, 35.40]	33.82±3.97 [32.78, 34.82]	30.35±2.98 [29.14, 31.29]	26.59±3.25 [25.43, 27.57]	<0.001	2·10^−5^
I1	41.58±9.87 [40.14, 45.43]	39.72±5.73 [37.89, 40.85]	36.74±5.29 [34.81, 38.63]	31.18±5.70 [29.31, 33.06]	<0.001	0.009
I2	27.26±4.72 [26.40, 28.92]	27.04±4.22 [26.06, 28.24]	23.58±3.49 [22.56, 25.07]	21.28±2.44 [20.59, 22.20]	<0.001	7·10^−5^

Measurements in μm

SD: Standard Deviation; CI: Confidence Interval; IPL: Inner plexiform layer

Given that the early diagnosis of glaucoma is a key factor, and with the objective of modifying the course of the disease, a more detailed analysis was performed to try and find the differences that mRNFL, GCL and IPL could present between OHT patients (Group 2) and early glaucomatous patients (Group 3) finding statistically significant differences in S1, S2, I1 and I2 sectors of mRNFL; N1, N2, T1, T2, S1, S2 I1 and I2 sectors of GCL; and N2, T1, T2, S1, S2 I1 and I2 sectors of IPL (Tables 1–3).

**Fig 2:**
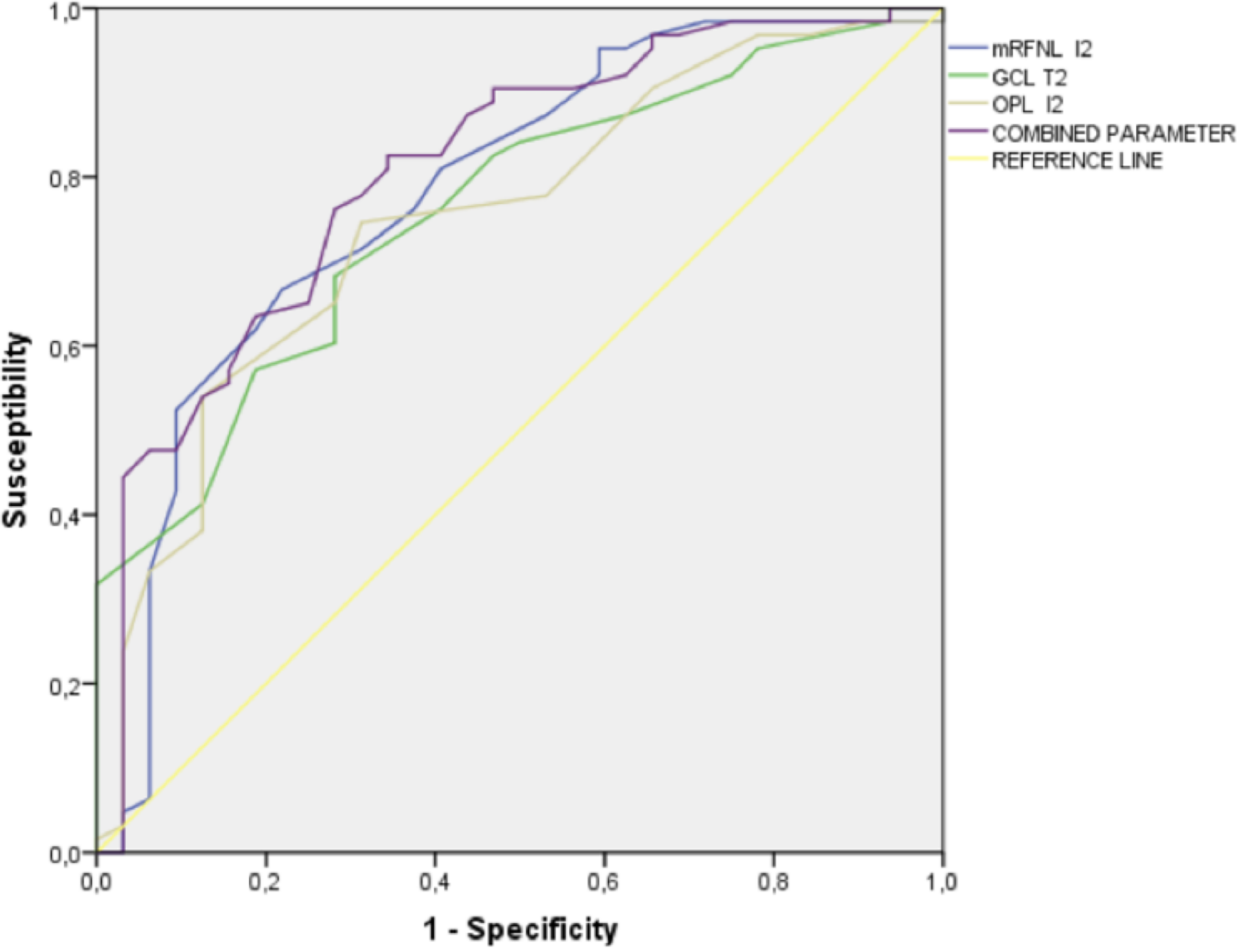
Receiver-operating characteristic (ROC) curve for early glaucoma diagnosis. Outer inferior sector of macular retinal nerve fiber layer (I2 mRNFL), outer temporal sector of ganglion cell layer (T2 GCL) and outer temporal of inner plexiform layer (T2 IPL) and a combination of all three.

Receiver-operating characteristic (ROC) curves were created in order to evaluate the diagnosis capacity of these results to discriminate between OHT and early glaucomatous damage using the sectors with the most significant differences for each layer (I2 for mRNFL, T2 for GCL and T2 for IPL) and a combination of all three (Fig 2).

Areas under the ROC curves were 0.781±0.052 for mRNFL I2, 0.760±0.050 for GCL T2, 0.767±0.049 for IPL T2 and 0.807±0.048 for the combination of all three, with a best combination of specificity/sensitivity of 76.2/71.9 and a cutoff value of 102.5 μm (Table 4). The advantages of using this combination are the greater sensitivity and specificity figures it shows, and the fact that it presents less risk of being affected by measurement errors in any of the three sectors.

**Table 4:** Areas under the curve and cutoff values for early diagnosis.

Parameter	P value	Area under the curve	80% Specificity Sensitivity	Cutoff value	90% Specificity Sensitivity	Cutoff value	Best combination Specificity Sensitivity	Cutoff value
mRNFI2	8·10^−6^	0.781±0.052	62	38.5	52.4	39.5	81/59.4	34.5
GCLT2	1·10^−5^	0.760±0.050	57.1	35.5	38.9	38.0	76.2/59.4	32.5
IPLT2	2·10^−5^	0.767±0.049	54	33	43	34	76.2/68.7	31.5
Comb	4·10^−7^	0.807±0.048	64	105	48	109.5	76.2/71.9	102.5

Measurements in μm

mRNFL: macular retinal nerve fiber layer; GCL: Ganglion cell layer; IPL: Inner plexiform layer; Comb: combination parameter

Receiver-operating curves to evaluate diagnosis capacity using the combined parameter to discriminate between groups are shown in S2 File.

## Discussion

Early diagnosis is one of the greatest challenges in glaucomatous pathology, as it is a progressive disease that is characterized by an irreversible loss of RGCs and RNFL [4]. The macular area is the most densely populated region by RGCs in the retina [34], several studies have shown that macular measurements with SD-OCT were as good as the pRNFL measurements in detecting glaucoma [18–23].

Zeimer et al [11] described macular thinning in glaucoma patients. Ishikawa et al [13] designed a new algorithm for macular segmentation to analyse all retinal layers. Statistically significant differences were found in the inner retinal macular complex (IRC: GCL + IPL + INL) between the healthy and the glaucomatous population. Those measures offered a similar diagnostic capacity to the study of the pRFNL in terms of discriminating between patients with glaucoma and healthy controls.

Developments in SD-OCT such as eye tracking, noise reduction, or B-scan averaging to further improve image quality have awakened the interest on the macula to study glaucomatous changes [35]. New protocols for the automated segmentation of retinal layers have been recently developed [36] therefore it is now possible to study each part of the ganglion cell: its axons are projected across the inner surface of the retina creating the mRNFL, the cell bodies form the GCL, and the dendrites constitute the IPL, so their study in different stages of the disease could be useful.

The focus of the analysis was set on the comparison between the OHT group and the early glaucoma group. Parameters combining several retinal layers grant greater area under the curve as well as better discrimination capacity, due to their ability to reduce the possibility of measurement errors in individual layers. The best single parameter to discriminate between OHT and glaucoma patients is mRFNLI2 with a cutoff value of 34.5 μm and a best combination of specificity/sensitivity of 81/59.4. These results are similar to those found by Hood [36] et al which describe that the macula is particularly susceptible to glaucomatous damage in this area.

Zangwill et al [37] found that several baseline topographic optic disc measurements were significantly associated with the development of POAG in Ocular Hypertension Treatment Study participants. Miglior et al [38] also found several baseline Heidelberg Retinal Tomograph (HRT) parameters significantly associated with the development of POAG among the European Glaucoma Prevention Study participants. More recently Colombo et al [39] showed that the individual risk to develop POAG within 5 years in OHT individuals is significantly correlated with pRNFL OCT parameters. Such studies tend to prove that there might be basal structural alterations in OHT patients. At the same time this data coincides with the results obtained in our procedure where we have found significant differences between healthy and OHT subjects regarding the mRFNL.

There is a sparse number of studies analysing the outer retinal layers in patients with glaucoma. Some of them show thinning at this level [24,28–30], whilst in other researches, as ours, no significant differences can be found between healthy and glaucoma subjects [25–27]. One of the limitations we have encountered in our research, is the measurement of the PRC by means of a formula in which the thickness of RPE layer was deduced from the total thickness (from outer limiting membrane to Bruch membrane). Due to the fact that this measurement is not direct, it cannot be considered as absolutely precise.

## Conclusions

To the best of our knowledge, this is the first study which analyses eight different retinal layers in the same patient, across 9 macular sectors, using Heidelberg's Spectralis OCT automated segmentation software in healthy population, OHT, early glaucoma and moderate-advanced POAG patients. There were no detected alterations of the outer retinal layers in any of the groups. At the same time, significant differences were found between healthy and ocular hypertensive patients, as well as in ocular hypertensive and early glaucoma patients at the inner retinal layers.

## Supporting information

S1 File Thickness values for all layers in each of the 9 ETDRS sectors. Healthy (Group 1), ocular hypertensive patients (Group 2), early glaucoma patients (Group 3) and moderate-advanced glaucoma patients (Group 4). Retinal nerve fiber layer (RNFL), ganglion cell layer (GCL), inner plexiform layer (IPL), inner nuclear layer (INL), outer plexiform layer (OPL), outer nuclear layer (ONL), photoreceptor layer (PRC) and retinal pigment epithelium (RPE). The average of all points within the inner 1-mm radius circle was defined as central foveal thickness (C0). The intermediate ring was divided into four sectors designated as inner superior (S1), inner nasal (N1), inner inferior (I1), and inner temporal (T1); and so was the outer ring, with four sectors designated as outer superior (S2), outer nasal (N2), outer inferior (I2), and outer temporal (T2).
(PDF)

S2 File Receiver-operating curves to evaluate the diagnosis capacity using the combined parameter. (PDF)

S3 File Database. (XLSX)
