# Supplementary material for: Analysis of inner and outer retinal layers using spectral domain optical coherence tomography automated segmentation software in ocular hypertensive and glaucoma patients
Source: PLoS One. 2018 Apr 19;13(4):e0196112. doi: 10.1371/journal.pone.0196112 (PMC5908140; doi:10.1371/journal.pone.0196112)
Supplement: S1 File — Healthy (Group 1), ocular hypertensive patients (Group 2), early glaucoma patients (Group 3) and moderate-advanced glaucoma patients (Group 4). Retinal nerve fiber layer (RNFL), ganglion cell layer (GCL), inner plexiform layer (IPL), inner nuclear layer (INL), outer plexiform layer (OPL), outer nuclear layer (ONL), photoreceptor layer (PRC) and retinal pigment epithelium (RPE). The average of all points within the inner 1-mm radius circle was defined as central foveal thickness (C0). The intermediate ring was divided into four sectors designated as inner superior (S1), inner nasal (N1), inner inferior (I1), and inner temporal (T1); and so was the outer ring, with four sectors designated as outer superior (S2), outer nasal (N2), outer inferior (I2), and outer temporal (T2). (PDF) [file pone.0196112.s001.pdf]

Figure 2.Thickness values for all layers in each of the 9 ETDRS sectors.

Healthy (Group 1), ocular hypertensive patients (Group 2), early glaucoma patients (Group 3) and moderate-advanced glaucoma patients (Group 4). Retinal nerve fiber layer (RNFL), ganglion cell layer (GCL), inner plexiform layer (IPL), inner nuclear layer (INL), outer plexiform layer (OPL), outer nuclear layer (ONL), photoreceptor layer (PRC) and retinal pigment epithelium (RPE). The average of all points within the inner 1-mm radius circle was defined as central foveal thickness (C0). The intermediate ring was divided into four sectors designated as inner superior (S1), inner nasal (N1), inner inferior (I1), and inner temporal (T1); and so was the outer ring, with four sectors designated as outer superior (S2), outer nasal (N2), outer inferior (I2), and outer temporal (T2).

C0

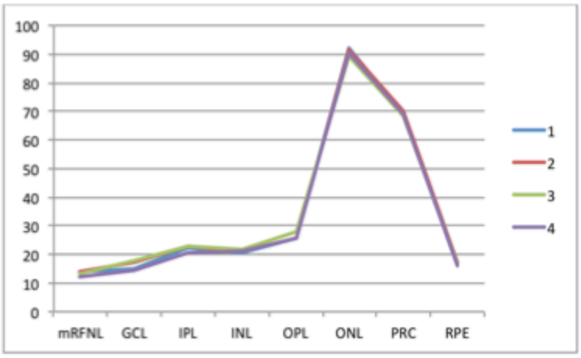
100
90
80
70
60
50
40
30
20
10
0
mRFNL
GCL
IPL
INL
OPL
ONL
PRC
RPE
1
2
3
4

N1

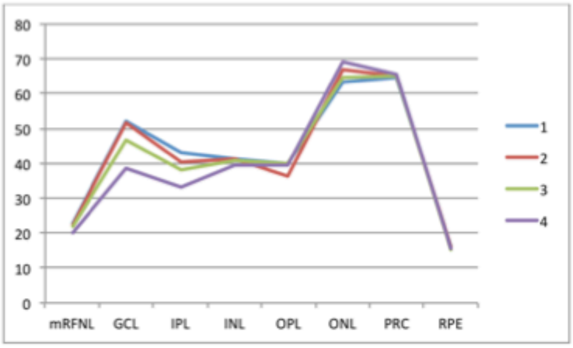
80
70
60
50
40
30
20
10
0
mRFNL
GCL
IPL
INL
OPL
ONL
PRC
RPE
1
2
3
4

N2

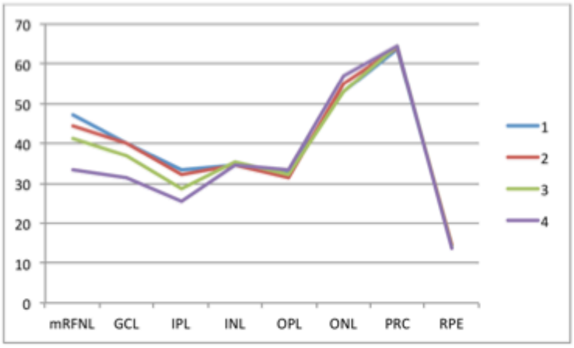
70
60
50
40
30
20
10
0
mRFNL
GCL
IPL
INL
OPL
ONL
PRC
RPE
1
2
3
4

S1

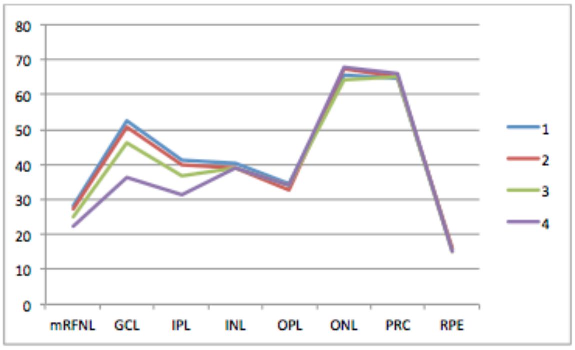
80
70
60
50
40
30
20
10
0
mRFNL
GCL
IPL
INL
OPL
ONL
PRC
RPE
1
2
3
4

S2

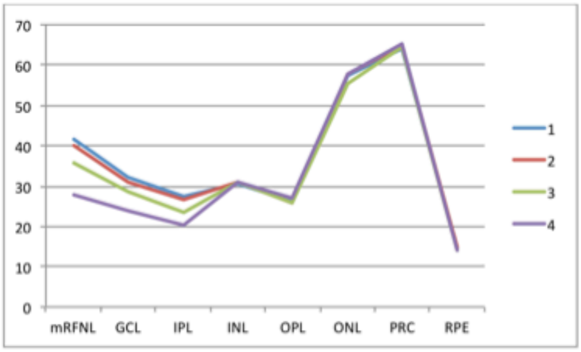
70
60
50
40
30
20
10
0
mRFNL
GCL
IPL
INL
OPL
ONL
PRC
RPE
1
2
3
4

T1

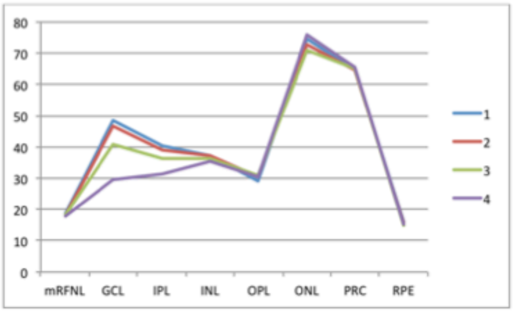
80
70
60
50
40
30
20
10
0
mRFNL
GCL
IPL
INL
OPL
ONL
PRC
RPE
1
2
3
4

T2

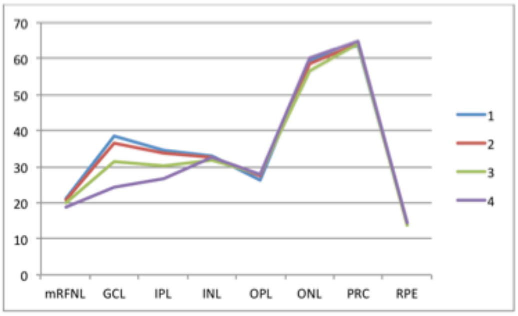
70
60
50
40
30
20
10
0
mRFNL
GCL
IPL
INL
OPL
ONL
PRC
RPE
1
2
3
4

I1

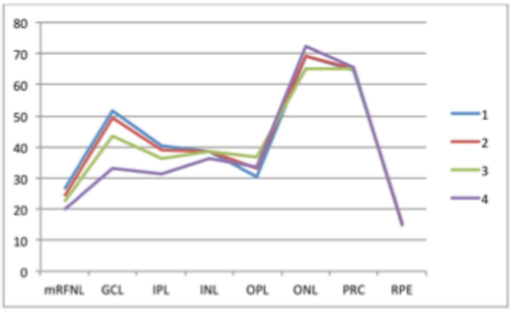
80
70
60
50
40
30
20
10
0
mRFNL
GCL
IPL
INL
OPL
ONL
PRC
RPE
1
2
3
4

I2

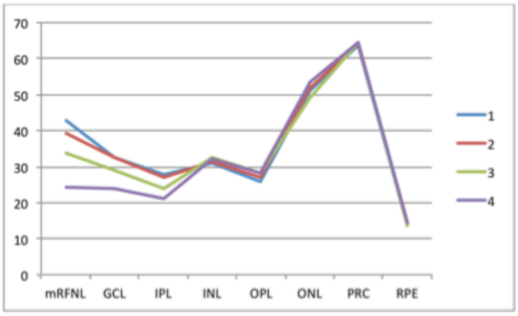
70
60
50
40
30
20
10
0
mRFNL
GCL
IPL
INL
OPL
ONL
PRC
RPE
1
2
3
4
