# Supplementary material for: Analysis of inner and outer retinal layers using spectral domain optical coherence tomography automated segmentation software in ocular hypertensive and glaucoma patients
Source: PLoS One. 2018 Apr 19;13(4):e0196112. doi: 10.1371/journal.pone.0196112 (PMC5908140; doi:10.1371/journal.pone.0196112)

S2 Fig. Receiver-operating curves to evaluate the diagnosis capacity using the combined parameter

Group 1(healthy) and 3(early glaucoma): 0.892±0.039

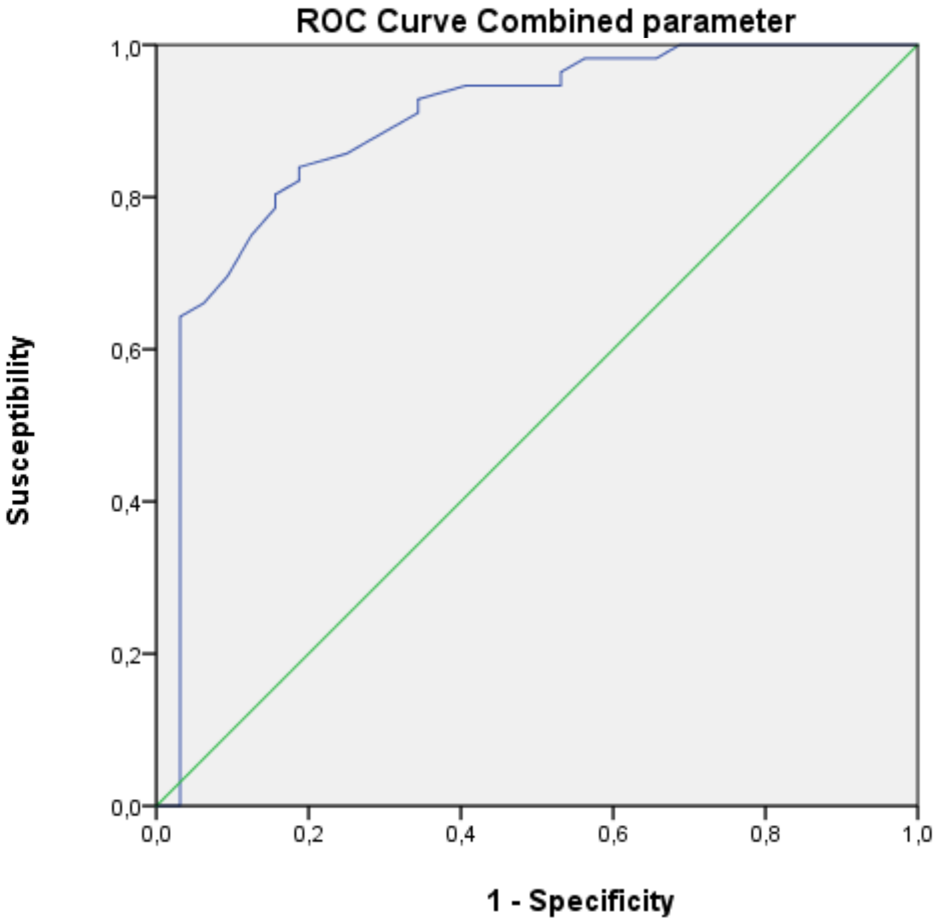

Group 1(Healthy) and 4 (moderate-advanced glaucoma): 0.992±0.06

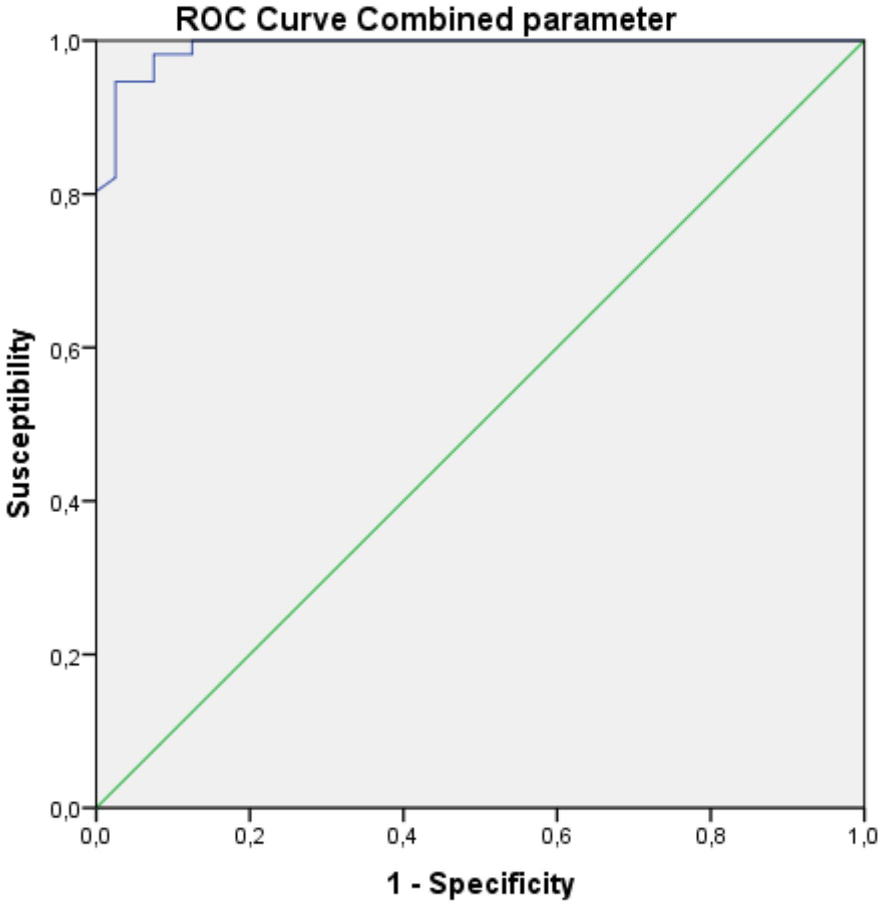

Group 2(OHT) and 4 (moderate-advanced glaucoma): 0.977±0.013

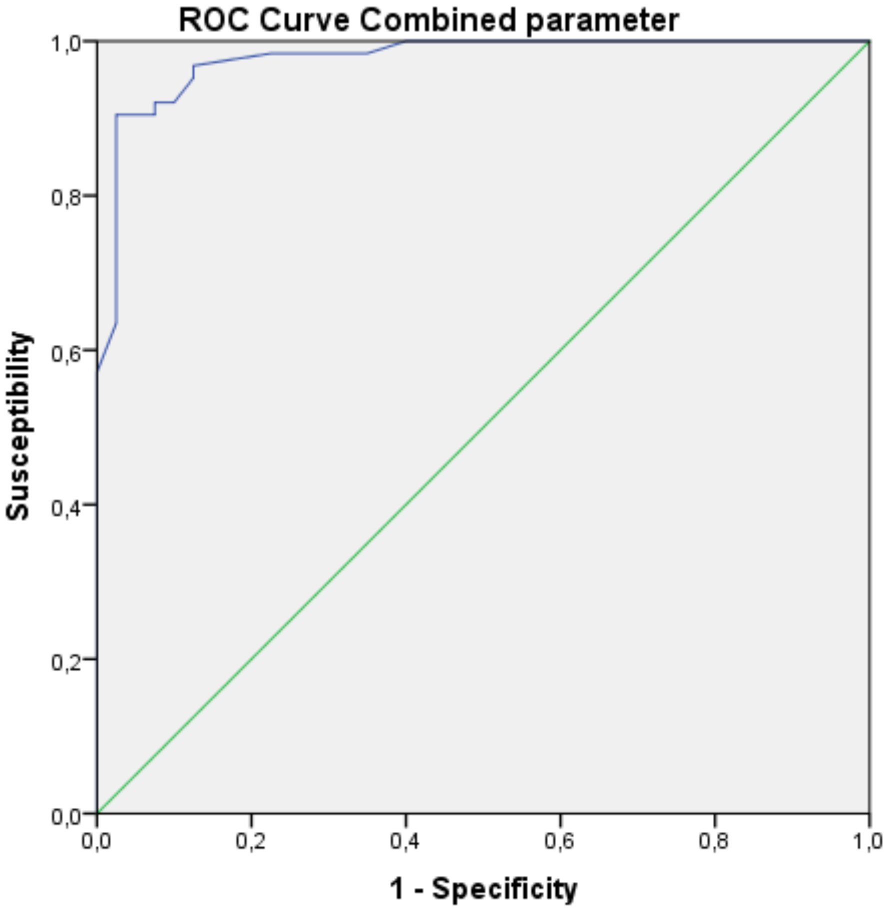

Group 3(early glaucoma) and 4 (moderate-advanced glaucoma):
0.878±0.40

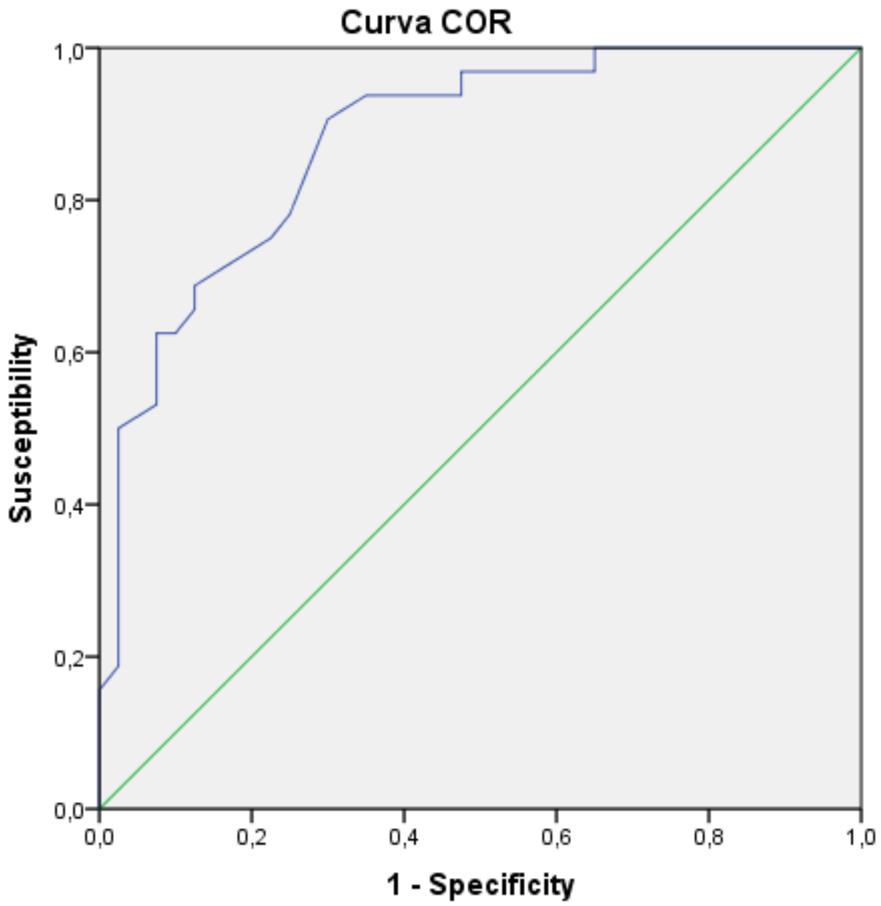

Supplement: S2 File — (PDF) [file pone.0196112.s002.pdf]
